# Coinfections Dampen the Effects of Temperature on Host–Parasite Interactions

**DOI:** 10.1111/ele.70464

**Published:** 2026-10-14

**Authors:** I. Will, C. A. Smith, T. E. Hector, K. C. King

**Affiliations:** ^1^ Department of Biology University of Oxford Oxford UK; ^2^ Department of Zoology University of British Columbia Vancouver Canada; ^3^ Department of Mathematical Sciences University of Bath Bath UK; ^4^ Department of Microbiology & Immunology University of British Columbia Vancouver Canada

**Keywords:** *Caenorhabditis elegans* nematodes, climate change, coinfection, emerging disease, *Leucobacter* bacteria, parasites, protection, temperature

## Abstract

Coinfections by multiple parasite species are widespread and often modify disease outcomes. To what degree other forms of ecological complexity, such as environmental change, influence the impacts of coinfecting parasites on disease remains unclear. We exposed nematodes ( *Caenorhabditis elegans* ) to naturally coinfecting parasites ( *Leucobacter celer* and *Leucobacter musarum*) at temperatures across the host's thermal range. While we found that host mortality varied widely with temperature during single infections, coinfections buffered hosts against temperature‐mediated variation via ongoing parasite competition. We extended our experiments with mathematical models indicating that changing virulence, rather than transmission, most plausibly underpins temperature‐dependent host mortality. At moderate, but not extreme temperatures, we found coinfection continued to dampen mortality outcomes due to another source of host variation, genotypic susceptibility. Coinfection may provide a stabilizing effect on disease in a warming world—but this effect can falter when hosts are faced with multiple stressors.

## Graphical abstract

Temperature can drive large shifts in host–parasite interactions, but these shifts need not correspond to strong effects on coinfecting parasite interactions. Coinfection may provide a stabilizing effect on disease in a warming world—but this effect can falter when hosts are faced with multiple stressors.

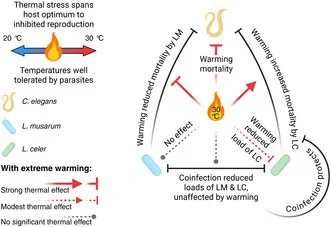

## 1 Introduction

Virtually all organisms host multiple parasite genotypes and/or species at some point in their lives. Interactions between parasites can determine the level of virulence (i.e., host harm due to infection) as coinfections often modify disease outcomes in ways not predicted by one‐host one‐parasite paradigms (Alizon et al. 2013; Balmer and Tanner 2011; Graham 2008; Hoarau et al. 2020; Pedersen and Fenton 2007; Tollenaere et al. 2016). Exploitative parasite competition (e.g., for host resources) or facilitation (e.g., by modification of the immune system) can worsen disease for coinfected hosts (Alizon et al. 2013; Cressler et al. 2016; Ramesh and Hall 2025), increasing host mortality (Cornet and Sorci 2010; Louhi et al. 2015; O'Keeffe et al. 2024) and parasite burdens (Lass et al. 2013; Susi et al. 2015). Alternatively, parasites can diminish each other's infectivity, thereby reducing the harmful effects of disease (Ford et al. 2016; Inglis et al. 2009; King et al. 2016). These ‘protective coinfections’ can be conferred by apparent (e.g., via host immunity), exploitative (e.g., for public goods), or interference (e.g., by toxin production) forms of competition (Alizon et al. 2013; Ashby and King 2017; Cressler et al. 2016; Ramesh and Hall 2025). However, wild host organisms are exposed to variable and extreme environmental conditions likely to alter host resistance and tolerance, as well as parasite growth and transmission, that underpin disease outcomes.

Global climate change is predicted to promote novel or alter existing host–parasite and parasite–parasite interactions, driving emerging disease (Carlson et al. 2022; Morales‐Castilla et al. 2021). The interaction of temperature and infection is often predicted to exacerbate disease during ‘thermal mismatches’ when the host experiences more severe thermal stress than their parasites (Cohen et al. 2017, 2020; Sauer et al. 2020). Ectothermic animal hosts may be most vulnerable (Cohen et al. 2020), while smaller parasites may often gain the upper hand due to their broader thermal tolerances (Rohr et al. 2018), but certainly with exception (Gehman et al. 2018). The interaction of single species infections and temperature modifies both disease outcomes (Cohen et al. 2020; Hector et al. 2022; Ismail et al. 2024; Paull and Johnson 2014; Ware‐Gilmore et al. 2021) and thermal performance (Bates et al. 2011; Hector et al. 2019, 2022; Hoang et al. 2019; Hudson et al. 2024; Martins et al. 2023). Underpinning these processes, at least in part, the metabolic theory of ecology connects the basic kinetics of metabolism to organismal traits and interactions, such a parasitism (Brown et al. 2004; Kirk et al. 2018). This generalization can offer reasoned baseline predictions for host–parasite interactions, but may not well capture idiosyncratic responses or cases at thermal extremes (Kirk et al. 2018). Similarly building from fundamental principles, dynamic energy budget frameworks consider how resources are allocated (i.e., tradeoffs) within an individual and can help explain disease outcomes in light of the uptake and expenditure of energy (Douhard et al. 2025; Erazo et al. 2025; Hall et al. 2009). Temperature and disease could affect host uptake (e.g., food availability, foraging effort) and/or expenditure (e.g., immunity, thermal stress response, reproduction and parasite consumption). However, these concepts have not been as widely and rigorously tested during coinfections, which are more relevant to wild disease ecology than single parasite infections (Telfer et al. 2010).

Environment, especially temperature, can modify coinfecting parasite prevalence (Fargues and Bon 2004; Friesen et al. 2021; Halle et al. 2024), distribution (Oakgrove et al. 2014), growth (Budischak et al. 2015; Vaumourin and Laine 2018) and competition (Alcaide et al. 2021; Greenrod et al. 2024). Coinfection dynamics can reflect temperature impacts on individual parasite species occurrence (Halle et al. 2024) or more complex interactions stemming from asymmetric competitiveness mediated by thermal performance (Greenrod et al. 2024). The relationship of coinfecting parasites, host and temperature has been shown to produce divergent virulence outcomes (Carpenter et al. 2024; Thomas et al. 2003) – although why, is not always known. Still unclear is if temperature dependent host–parasite effects modify coinfecting parasite interactions, thereby altering disease outcomes.

We tested whether coinfection interactions were robust across temperatures, employing experiments and mathematical models to identify the processes governing these responses. Alternatively, temperatures that impact hosts could impact coinfection interactions, altering disease outcomes. To test these hypotheses, we used naturally coinfecting parasites at temperatures representative of their source locality, covering a range of host thermal stress. We leveraged a nematode host ( *Caenorhabditis elegans* ) and two bacterial parasites ( *Leucobacter celer* and *Leucobacter musarum*) co‐isolated from a wild congener host, *Caenorhabditis tropicalis* (Bates et al. 2021; Clark and Hodgkin 2015). In *C. elegans* , *Leucobacter*‐infected host mortality has been observed to vary across temperatures (*L. musarum*), moisture levels ( *L. celer* ) and host genotypes, and have tradeoffs between resisting either parasite (Bates et al. 2021; Hector et al. 2025; Hodgkin et al. 2013; Loer et al. 2015; O'Rourke et al. 2023; Parsons et al. 2014). *Leucobacter celer* attenuates or limits *L. musarum* infections during coinfection, in the absence of extreme thermal stress (Bates et al. 2021; Hodgkin et al. 2013). We assessed host mortality, fecundity and parasite load across combinations of infections and temperatures spanning the host's thermal optimum to upper reproductive limit. Thermal mismatches and metabolic theory would predict increasing parasite fitness moving from the lower host optimum temperature towards higher thermal stress for hosts, when these temperatures are well tolerated by the parasites (as in our experiments) (Cohen et al. 2020; Kirk et al. 2018). However, substantial changes in host energy budgets and physiology as a result of extreme thermal stress (i.e., castration) (Hall et al. 2009), may lead to deviations from those predictions. Based on these initial principles, we would predict that the fundamental relationship between temperature and disease to be similar for the congener parasite species tested. We further tested if parasite interactions were robust to direct (genetic) modification of host condition. Using mathematical models, we extended our experimental results to determine how transmission and virulence could explain temperature‐dependent mortality, while remaining consistent with the effects of coinfection. Taken together, we tested how coinfection shapes disease when temperature radically alters host–parasite interactions.

## 2 Methods

### 2.1 Study System

We used isogenic N2 strain *Caenorhabditis elegans* nematodes as hosts in all experimental assays. For mortality assays, we also used *srf‐2* (*yj262*) mutant *C. elegans* (Politz et al. 1990). These *srf‐2* mutants have a missense mutation in the *srf‐2* gene (F59C6.8) producing cuticular glycosylation defects that impact locomotion and infection susceptibility (see below), but otherwise have near normal growth (O'Rourke et al. 2023). We maintained *C. elegans* on nematode growth medium plates (NGM) colonized by *Escherichia coli* (OP50) as a food source (Stiernagle 2006).

The two naturally co‐isolated bacterial parasites we used were *Leucobacter celer* subsp. *astrifaciens* (CBX151) and *Leucobacter musarum* subsp. *musarum* (CBX152) (Clark and Hodgkin 2015). *Leucobacter* infections cause disease in both the wild host *Caenorhabditis tropicalis* (Silva et al. 2026) and *C. elegans* (Hodgkin et al. 2013). *Leucobacter musarum* kills *C. elegans* in a temperature‐dependent manner (Hector et al. 2025; Hodgkin et al. 2013). *Leucobacter celer* infection is essentially sub‐lethal at those temperatures, but virulence has been observed as increased host generation times (Hodgkin et al. 2013) and gene expression indicating immunity‐reproduction fitness tradeoffs (Will et al. 2025). Host tradeoffs between resisting one or the other parasite have been documented for *C. elegans* mutant lines (e.g., *srf‐2*) (Bates et al. 2021; Hodgkin et al. 2013; Li et al. 2025).

To generate working cultures of *Leucobacter*, we inoculated 3 mL Lennox lysogeny broth (LB) from glycerol freezer stocks and incubated them at 25°C for 24 h with shaking. For OP50, we instead inoculated 30 mL of LB. We confirmed all cultures were within optical density (OD, 630 nm) 0.2–0.6 before proceeding with assay protocols. All OD measurements were taken in 96‐well plates with 200 μL volumes with a Varioskan Lux plate reader (Thermo Scientific).

### 2.2 Parasite Thermal Optima

We estimated optimal growth temperatures for the parasites by combining *in silico* predictions and in vitro growth. We used Tome (v. 1.0.0) (Li et al. 2019) and its pre‐trained machine learning model on proteome sequence data (Will et al. 2025) from both *Leucobacter* species to generate *in silico* predictions. The predictions produced were single point estimates of the optimal temperature. For growth assays, we standardized cultures to OD 0.2 ± 0.02. Each biological replicate was a well‐plate containing LB‐only blanks (*n* = 12 wells) and *L. celer* and *L. musarum* inoculated samples (*n* = 24 wells per species). We filled inoculated wells with 200 μL of a 1:10 dilution of initial OD 0.2 cultures. Plates were then incubated with gentle shaking (60 rpm, ‘medium force’), with an OD reading every 15 min, for 24 h.

We conducted growth curve assays at 20°C, 25°C and 30°C (*n* = 3 plates per temperature). The *Leucobacter* species were originally collected from Cape Verde, where soil temperatures range approximately 15°C–30°C (Hector et al. 2025; Lembrechts et al. 2022). This temperature range includes thermal signposts for the study organisms: the optimum (20°C), maximum (25°C) and slightly beyond (30°C) for *C. elegans* multi‐generational fertility (Begasse et al. 2015), with some indication that the *Leucobacter* optimum is near 30°C (Clark and Hodgkin 2015). We analysed growth curve data using gcplyr (v. 1.10.0) in R to estimate maximum growth rates per well on ‘median then average’ smoothed data (window = 7 data points for each) (Blazanin 2024).

### 2.3 Infection Mortality Assays

We grew age‐synchronized *C. elegans* using a bleaching protocol (Stevens et al. 2025). We plated approximately 1500 larval stage 1 nematodes to 9 cm food plates and grew them at 20°C for 48 h, until they were young adults, for subsequent experimentation. Working cultures of bacteria were used to create infection assay plates of four kinds: OP50 only (control), *L. celer* (‘LC’ treatment), *L. musarum* (‘LM’ treatment), or both bacterial parasites (coinfection). All infection assay plates (6 cm NGM plates) received 80 μL of OP50 at OD 0.3 to feed the nematodes. For LC, LM and coinfection, we additionally added 20 μL per target *Leucobacter* species (OD 0.2), ensuring single‐infection and coinfection treatments had the same inoculum per species. All plates incubated at 25°C for 18 h to grow the bacteria. We then transferred approximately 100 young adult nematodes to each plate to begin the infection assay.

Our experimental design was fully factorial, combining infection and temperature (Figure S1). Nematodes from each infection treatment were placed at one of four temperature regimes for 30 h: 20°C, 25°C, 30°C, or ‘heatwave’. Heatwave plates incubated at 25°C (12 h), 35°C (4 h) and 25°C (14 h). For Cape Verde, 35°C soil temperatures have been recorded but are above average—suggesting transient heatwave exposures are realistic (Hector et al. 2025). For *C. elegans* N2 mortality assays, we conducted seven independent batches, each with two replicates (assay plates with 100 nematodes) per treatment (*n* = 14 per treatment combination). To measure host mortality, we examined nematodes under a microscope; dead nematodes did not respond to gentle prodding by a wire. We performed mortality counts aseptically for downstream host fecundity and parasite load assays.

To test coinfecting parasite interactions in hosts with fundamentally altered susceptibilities, we used mutant *srf‐2* *C. elegans* (see Study System, above). These mutants allowed us to manipulate host–parasite relationships without directly altering parasite–parasite relationships. For *srf‐2* *C. elegans* , we conducted four batches of mortality assays, one with four replicates and the rest with two replicates per treatment (*n* = 10 per treatment combination).

### 2.4 Parasite Load and Host Fecundity Assays

Using a subset of the *C. elegans* N2 infection assay batches, we quantified parasite loads (colony forming units, CFUs) (5 batches, *n* = 10 per treatment) and host fecundity (3 of the batches used for parasite load batches, *n* = 6 per treatment) (Figure S1). After scoring mortality, we used a wire to pick 13 live nematodes per plate and transferred them to tubes with M9 buffer (Stiernagle 2006) supplemented with 0.1% Triton‐X‐100 (TX). We washed the nematodes three times in M9‐TX and suspended them in 200 μL of M9. As *Leucobacter* adheres to the host surface (Hodgkin et al. 2013), we did not surface‐sterilize the nematodes (Figure S2). Of the 13 washed nematodes, 10 individuals were used for CFU assays (see below) and three for fecundity assays (Appendix S1).

We estimated *Leucobacter* loads by counting bacterial CFUs from infected hosts. We selected live nematodes as we aimed to capture parasite loads during infection, instead of consumption of dead individuals. Although these survivors may have avoided or tolerated infection to a greater degree than their dead counterparts, they permitted consistent comparisons across treatments. Using 10 washed nematodes picked from infection plates (see above), we added zircon beads (0.5 mm) to the nematode suspension and homogenized samples (Qiagen TissueLyser II, 5 min, 30/s frequency). We serially diluted samples to 1:1000 in M9. We spotted three 10 μL technical replicates of each diluted sample onto LB agar plates and incubated them at 25°C for 48 h. Colony size and colour between *L. celer* , *L. musarum* and OP50 are distinct and can be distinguished by eye. We recorded the number of parasite CFUs per 10×‐nematode pool and calculated the average per nematode. We verified that environmental competition (co‐culture on assay plates) was likely only a minor influence on host bacterial load by measuring *Leucobacter* CFUs from infection assay plate lawns (Figure S3).

### 2.5 Statistical Models

We used generalized linear mixed models (GLMMs) to identify how infection and temperature may drive host mortality, host fecundity and bacterial load. For modelling continuous responses (bacterial load and host fecundity), we used frequentist negative binomial GLMMs (*p* ≤ 0.05 threshold). We used Bayesian logistic regressions to analyse host mortality data, which had more complicated data structures. We evaluated treatment effects by their posterior 95% credible intervals (95% CI). Informed priors for Bayesian mortality models reflected the range of mortality outcomes previously reported for *Leucobacter* infection of *C. elegans* (Bates et al. 2021; Hector et al. 2025) – ‘normal(−2, 3)’ for the intercept and all treatment categories with the default ‘student_t(3, 0, 2.5)’ for random batch effects (logit scale). For all statistical models, we considered temperature (levels: 20°C, 25°C, 30°C, and heatwave) and infection (levels: control, LC, LM and coinfection) as categorical fixed effects and included experimental batch as a random intercept effect. We reported a Bayes R^2^ for Bayesian models and a Nakagawa's pseudo‐R^2^ for frequentist models. We conducted all statistics with R (v. 4.4.0) in RStudio (v. 2023.12.1) (R Core Team 2022; RStudio Team 2020). We used R packages brms (v. 2.21.0) for Bayesian models, glmmTMB (v. 1.1.9) for frequentist models, and performance (v. 0.11.0) to assess models (Brooks et al. 2017; Bürkner 2021; Lüdecke et al. 2021). To extract predictions from model fits, we used brms function conditional_effects including random effects for the mortality models and package ggeffects (v. 2.3.2) (Lüdecke 2018) function predict_response for the CFU models. We also used R package ggplot2 (v. 3.5.1) and Inkscape software (v. 1.3.2) to produce and finalize figures (Wickham 2016).

### 2.6 Mathematical Models

We used mathematical modelling to understand how virulence and transmission could drive divergent host disease outcomes across conditions (e.g., temperature). Our models allowed changes in both transmission and virulence to explain population‐level mortality. Transmission described the transition from a susceptible host to infected host, rather than parasite accumulation or load. Virulence was mortality caused by infections. Rather than directly modelling temperature, our models described general‐case, non‐linear shifts in mortality, exemplified by those we observed from different temperature by infection interactions. We first described how virulence and transmission can shift population‐level mortality due to one parasite species in the environment (Figure 1; Appendix S2). This virulence‐transmission ‘mortality landscape’ was then constrained by the effect of coinfection. We considered hypothetical exclusion (transmission‐reducing) and attenuation (virulence‐reducing) mechanisms of protection (Figure 1). Coinfection was required to produce intermediate mortality (i.e., protection) to align with our empirical findings, thus constraining valid virulence‐transmission values initially established by single infections.

**FIGURE 1:**
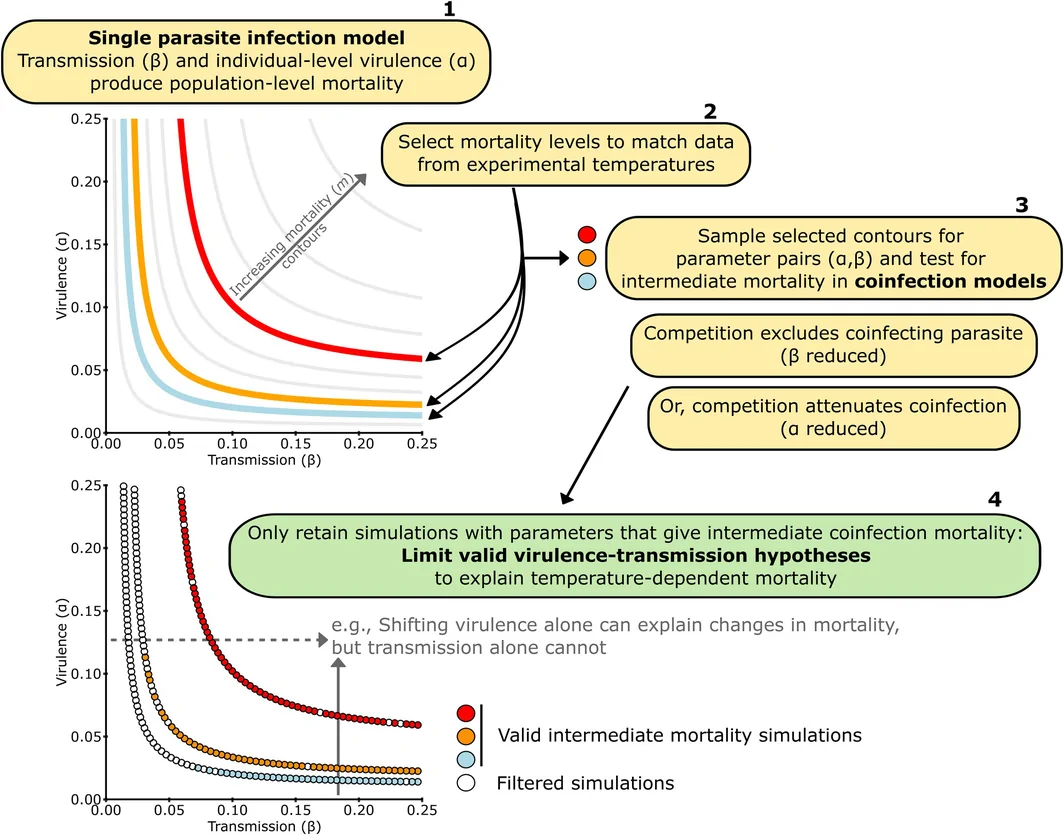
Coinfection was used to constrain single‐infection simulations of host population mortality to understand possible temperature‐virulence‐transmission relationships. The initial mortality contours (each contour represents some mortality level, *m*) for a single infection created a theoretical mortality landscape that established the fundamental ways that transmission (β) and virulence (α) can drive population‐level mortality (1). Contours farther from the axes indicate higher levels of mortality. We extracted virulence‐transmission parameter pairs along selected contours, chosen to reflect patterns in our experiments (2). We then tested those parameter pairs in coinfection models of either exclusion (β reduced by $y_{e}$) or attenuation (α reduced by $y_{a}$) (3). We required that valid simulations produced intermediate mortality during coinfection (an effect found in our experiments) (4). The remaining truncated virulence‐transmission contours constrained viable paths to move between mortality levels. Additional details can be found in Appendix S2.

The theoretical host population could be infected by two parasites, indexed as j=1,2, either singly or in coinfection (Appendix S2). The parasites were environmentally transmitted (as in our experiments) at a flat rate $\beta_{j}$ (transmission) and caused mortality at rate $\alpha_{j}$ (virulence) in infected hosts. The simulation timeframe was shorter than host generation time (i.e., no births). Non‐disease mortality was included as rate d. Coinfecting parasite competition produced intermediate mortality by either exclusion (reducing β) or attenuation (reducing α). The model did not distinguish if virulence attenuation might be driven by either resistance or tolerance. Exclusion was encapsulated by functions $E_{1}\left(y_{e1}\right)$ (resp. $E_{2}\left(y_{e2}\right)$) for varying exclusion by parasite 1 (resp. 2) against parasite 2 (resp. 1) with strength $y_{e1}$ (resp. $y_{e2}$). The strength of exclusion was $y_{\mathrm{ej}}\in \left[0,1\right]$, where 0 is no exclusion and 1 is complete exclusion. Alternatively, we considered attenuation, given as functions $A_{1}\left(y_{a1}\right)$ (resp. $A_{2}\left(y_{a2}\right)$), following the same naming convention. We defined $N_{0}$ as the initial population size, $S\left(t\right)$ as the susceptible population, $I_{1}\left(t\right)$ and $I_{2}\left(t\right)$ as the singly‐infected populations, and $C\left(t\right)$ as the coinfected population, after time t, in these ordinary differential equations:

(1) $$\frac{\mathrm{dS}}{\mathrm{dt}}=-\left(d+\beta_{1}+\beta_{2}\right)S,S\left(0\right)=N_{0,}$$

(2) $$\frac{dI_{1}}{\mathrm{dt}}=\beta_{1}S-\left(d+\alpha_{1}+\left(1-y_{e1}\right)\beta_{2}\right)I_{1},I_{1}\left(0\right)=0,$$

(3) $$\frac{dI_{2}}{\mathrm{dt}}=\beta_{2}S-\left(d+\alpha_{2}+\left(1-y_{e2}\right)\beta_{1}\right)I_{2},I_{2}\left(0\right)=0,$$

(4) $$\frac{\mathrm{dC}}{\mathrm{dt}}=\left(1-y_{e1}\right)\beta_{2}I_{1}+\left(1-y_{e2}\right)\beta_{1}I_{2}-\left(d+\left(1-y_{a2}\right)\alpha_{1}+\left(1-y_{a1}\right)\alpha_{2}\right)C.\;C\left(0\right)=0.$$

Mortality, $m=M\left(T\right)$, was the total proportion of the initial proportion that has died at the final time, T (Appendix S2). When testing the effect of exclusion or attenuation, the effect of the other was set to zero for simplicity.

We used these models for simulations (Appendix S2). Time T was fixed arbitrarily to the same time in all simulations. For each temperature (i.e., mortality level), we sampled 1000 parameter combinations for parasites 1 and 2 $\left(\beta_{1},\alpha_{1},\beta_{2,}\;\alpha_{2}\right)$. Per parameter combination, we then sampled 20 protection values $\left(y_{e1},y_{e2}\right)$ or $\left(y_{a1},y_{a2}\right)$ uniformly at random from the unit square, and calculated coinfection mortality (m). Using these coinfection mortalities, we filtered simulation results to require intermediate coinfection mortality. By plotting the accepted simulations (Figure 1), we ascertained how virulence and transmission could change across conditions (temperature) to produce mortality shifts exemplified by the patterns we observed experimentally. The model, equations and simulations are detailed in Appendix S2.

## 3 Results

### 3.1 Parasites Had a Higher Thermal Tolerance Than The Host

Genomic predictions and in vitro growth curve assays indicated both parasites well tolerate 20°C–30°C temperatures. Our computational analysis of the *Leucobacter* proteomes predicted thermal optima of 29.2°C ( *L. celer* ) and 28.9°C (*L. musarum*). Corroborating those findings empirically, we measured the highest maximum growth rate for both species at 30°C (2.3 and 2.0 h median doubling times for *L. celer* and *L. musarum*, respectively) as opposed to cultures grown at 20°C or 25°C (2.8–2.9 h doubling times) (Figure S4).

### 3.2 Parasite Fitness Was Reduced by Coinfection Across Temperatures

To test whether temperature and coinfection interactions modified parasite fitness, we modelled parasite fitness (CFU loads) as ‘Load ~ Temperature*Infection + (1|Batch)’ (Figure 2; Tables S1 and S2). We created two such frequentist GLMMs, one for *L. celer* and one for *L. musarum* loads, where the infection term could be either single or coinfection.

**FIGURE 2:**
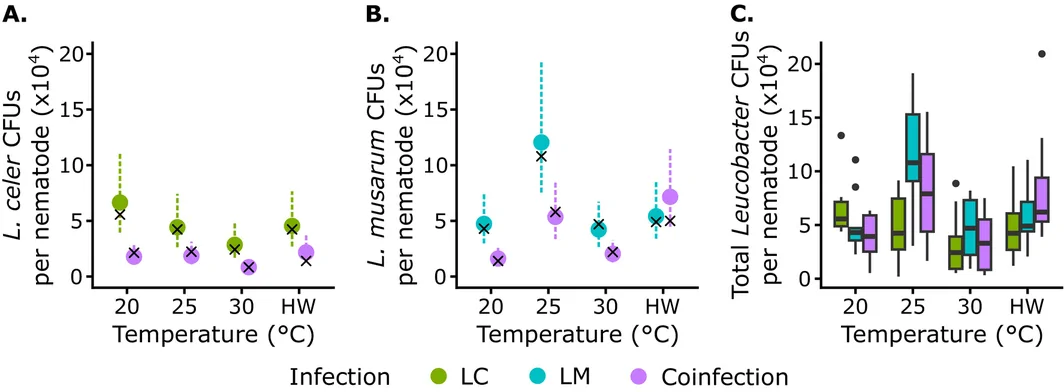
Parasite fitness (CFU load) was generally reduced by coinfection, with secondary effects of temperature. Load reductions during coinfection were larger than observed during in vitro co‐culture on assay plates alone (Figure S3). Per nematode values were calculated from 10‐nematode replicate pools. (A) *Leucobacter celer* load was reduced by high temperature (30°C) and by coinfecting *L. musarum* across temperatures. (B) *Leucobacter musarum* load peaked at 25°C and was typically suppressed by coinfection. However, during heatwave, *L. musarum* loads were not reduced by coinfection. (A, B) Points are predicted values from the GLMM with frequentist 95% confidence intervals as dashed lines (rather than Bayesian credible intervals reported for mortality models). Median values of the raw data (Figure S5) are given by crosses. (C) The boxplots show observed total parasite loads (i.e., coinfection treatments sum *L. celer* and *L. musarum* CFUs). Although coinfection reduced loads of both parasite species individually (A and B), the total *Leucobacter* load on coinfected hosts was not clearly below the lowest single‐infection load at any given temperature (C).

*Leucobacter celer* loads were reduced at high temperature and by coinfection with *L. musarum* (Figure 2A; Figure S5; Table S1). Corresponding to peak LC treatment mortality (see below), sustained high temperature at 30°C reduced *L. celer* loads during single infection (effect = −0.85, *z* = −2.89, *p* = 0.004). Coinfection further lowered *L. celer* loads at all temperatures, with a 61% reduction at 20°C (effect = −1.32, *z* = −4.48, *p* < 0.001). We did not detect a clear interaction of coinfection and temperature, indicating that the strength of parasite competition on *L. celer* was not sensitive to warming (Figure S8; Table S1).

*Leucobacter musarum* loads increased with moderate warming and typically decreased in coinfection (Figure 2B; Figure S5; Table S2). LM treated hosts had larger parasite loads at 25°C (effect = 0.93, *z* = 3.54, *p* < 0.001) (Figure 2B; Table S2), where we observed peak LM host mortality (Figure 3). *Leucobacter musarum* abundance decreased during coinfection at most temperatures, with a 67% reduction at 20°C (effect = −1.07, *z* = −4.10, *p* < 0.001) (Figure 2B; Table S2). However, during heatwave, *L. musarum* loads were not reduced by coinfecting *L. celer* (Figure 2B; Figure S8; Table S2). This change in underlying competition dynamics led to coinfection maintaining total parasite loads at or above the highest single‐infection during heatwave (Figure 2C). At all other temperatures, coinfection had loads near or slightly above the lowest single‐infection loads (Figure 2C).

### 3.3 Coinfection Dampened the Impacts of Temperature on Disease Outcomes

To test to what extent temperature and infection predicted host mortality, we constructed a Bayesian logistic regression: ‘Mortality ~ Temperature*Infection + (1|Batch)’ (Figure 3A; Table S3). We additionally constructed infection‐specific GLMMs of host mortality using parasite load and temperature predictors (Figure 3B; Appendix S3).

**FIGURE 3:**
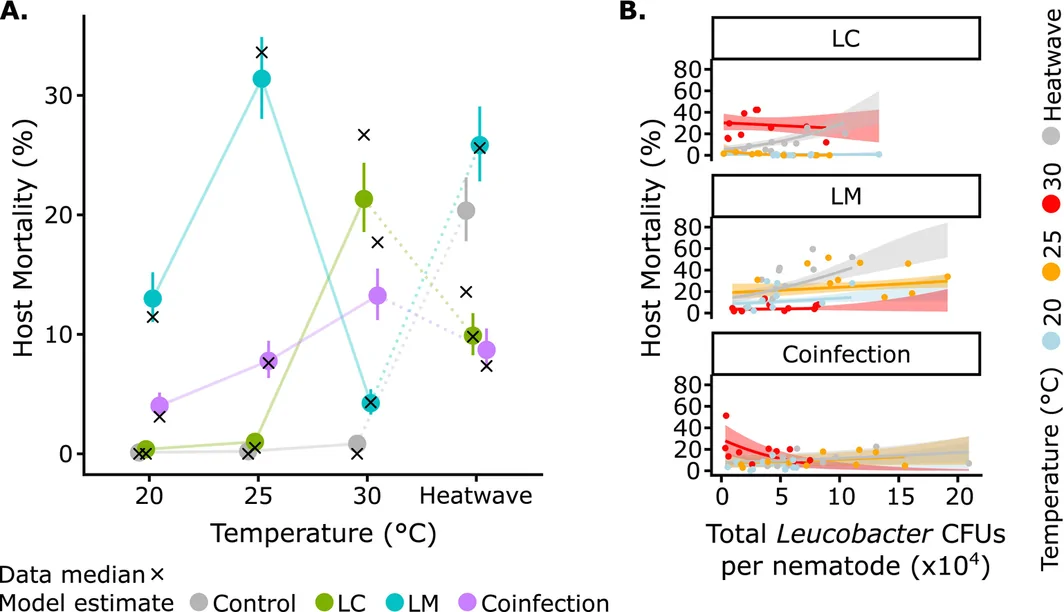
Coinfecting parasite interactions modified temperature‐dependent host mortality. (A) Model estimates (points) and 95% CI (bars) show LM (*L. musarum* alone) and LC ( *L. celer* alone) treatments with extreme warming (30°C) reversed in relative severity. However, at all temperatures, coinfection protected hosts from peak mortality of the most harmful single infection. Median values of the raw data (Figure S6) are given by crosses, typically laying within the 95% CI from model predictions. Points are connected by solid lines to show trends between constant temperatures, with a dashed connector for the heatwave treatment. (B) Parasite loads and host mortality did not have consistent, strong trends correlating *Leucobacter* CFUs and host death. Only LM treatments showed positive relationships between load and mortality, although these effects were not very strong (Appendix S3). LC infections during heatwave did not have sufficient statistical support to link load and mortality, despite an apparent positive slope (Appendix S3). Plotted points are the observed data, model estimates are given by the solid line with 95% CI shaded ribbons. The estimate solid lines are truncated at the maximum observed CFU value per treatment.

Host mortality varied in a parasite species and temperature‐dependent manner (Figure 3A; Figure S6; Table S3). We observed the clearest differences between moderate (20°C–25°C) and high (30°C) temperature. At both 20°C and 25°C, *L. celer* alone promoted negligible host mortality (0% and 0.5% LC median mortality, respectively) while *L. musarum‐only* infections worsened with mild warming (11% and 34% LM median mortality, respectively) (Figure 3A; Table S3; Data S1) (Bates et al. 2021; Hector et al. 2025; Hodgkin et al. 2013). When temperature increased to 30°C, we found opposing temperature‐by‐infection interactive effects between LC and LM treatments (Figure 3A; Figure 4; Table S3). LC treatment markedly increased host mortality relative to controls at 30°C (27% median mortality). Conversely, the LM treatment mortality was much reduced (4% median mortality) (Hector et al. 2025).

**FIGURE 4:**
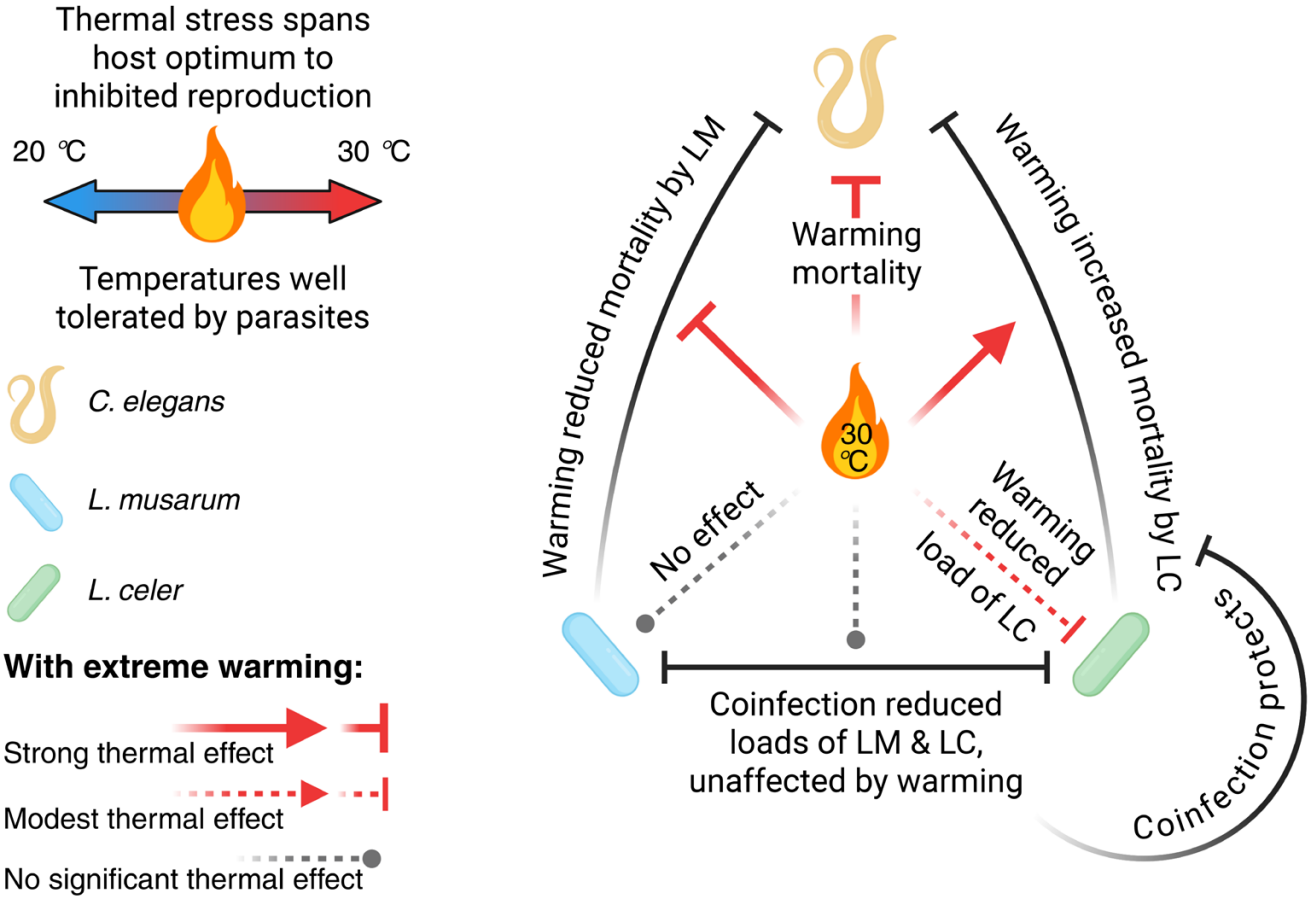
Extreme heating revealed thermally sensitive and thermally robust interactions that reflect host and parasite thermal tolerance. When hosts experience extreme thermal stress (30°C), disease outcomes change significantly, but with contrasting parasite‐specific effects. Parasite–parasite coinfection interactions continued to be competitive and provide protection. Parasite coinfection interactions thus dampened the possible effect of thermal stress on disease. Arrows indicate a causative link (e.g., coinfection producing intermediate mortality) or an increased effect (e.g., warming increased mortality from *L. celer* ). Bars show harm (e.g., coinfection reduces parasite loads) or a reduced effect (e.g., warming reduced mortality from *L. musarum*).

Across temperatures, coinfection resulted in host protection (Figure 3A). *Leucobacter celer* protected hosts against *L. musarum* during 20°C, 25°C, and heatwave treatments (Figure 3A; Table S3; Data S1). At 30°C, where LC infection was most lethal, coinfection by *L. musarum* was also protective, albeit to a lesser degree (Figures 3A and 4; Table S3; Data S1). This smaller protective effect at 30°C corresponded to a 34% mortality reduction, compared to 71%–77% reductions conferred by *L. celer* at other temperatures (Figure 3A). We also measured a sub‐lethal aspect of host fitness, host fecundity. Coinfection continued to partially protect hosts, buffering fecundity to intermediate levels. The fecundity assay methods and results are detailed in Appendix S1.

All infections caused less mortality during heatwave than would be predicted by their effect at 20°C (Figure 3A; Table S3). Heatwave drove the highest levels of mortality in uninfected *C. elegans* (13.6% median mortality). This increased ‘baseline’ accounted for much of the 25.6% median mortality during LM‐heatwave treatments. The LM by heatwave interaction reflected a significant reduction of the mortality effect from infection compared to 20°C LM (Table S3). Infections including *L. celer* (LC or coinfection) were avirulent, or possibly, protected against heatwaves (Table S3; Data S1). Although protection against thermal stress is biologically plausible, given that the median observed control‐heatwave mortality lies much closer to LC or coinfection than the model estimate (Figure 3A), we leave this possible effect for future validation.

LC infections with higher loads did not increase mortality, whereas LM infections likely did (Figure 3B; Appendix S3). Coinfection load only had a temperature specific effect at 30°C (Figure 3B; Appendix S3). At 30°C, total coinfection loads reduced host mortality (Figure 3B)—however, underlying this observation was that the two species had opposing impacts on mortality (Figure 3; Appendix S3). A coinfected host mortality GLMM using load predictors per species revealed load‐temperature interactions at 30°C: a positive relationship between host mortality and *L. celer* loads (effect = 0.93, 95% CI = 0.39, 1.47) was counteracted by a negative relationship with *L. musarum* (effect = −1.84, 95% CI = −2.71, −0.96) (Appendix S3).

### 3.4 Coinfections Continued to Buffer Disease Outcomes in Hosts With Altered Susceptibility

We tested if coinfection protection persisted despite changes to host–parasite interactions across contexts other than temperature. We used *srf‐2* mutant *C. elegans* that have inversed susceptibilities to the *Leucobacter* parasites (Hodgkin et al. 2013; Li et al. 2025), thus directly changing host–parasite interactions. We created a GLMM of *srf‐2* host mortality, in the same form as that used for N2 host data: ‘Mortality ~ Temperature*Infection + (1|Batch)’ (Figure 5; Table S4).

**FIGURE 5:**
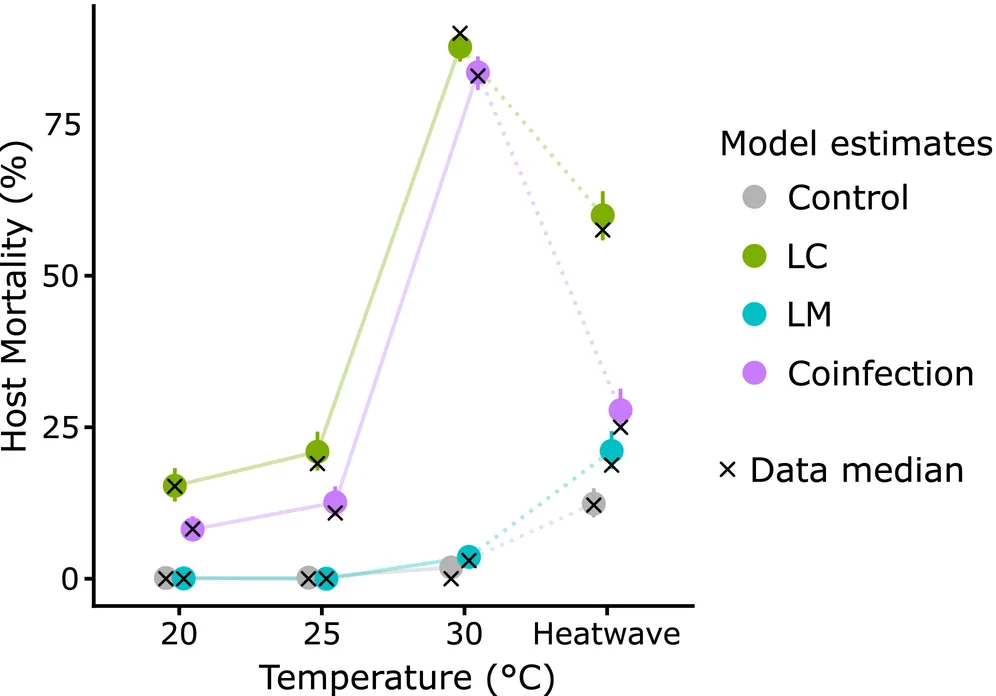
Parasite interactions continued to produce intermediate mortality in genetically susceptible hosts, except with the temperature that drove the highest virulence. Model estimates (points) and 95% CI (bars) show LM (*L. musarum* alone) and LC ( *L. celer* alone) have inversed mortality outcomes compared to N2 genotype hosts at lower temperatures and exacerbated LC mortality at higher temperatures (Figure 3). However, parasite competition was still capable of protecting *srf‐2* mutant hosts, leading to intermediate mortality. This protection was notably less effective with combined thermal stress and genetic susceptibility (LC, *srf‐2* and 30°C combination). Single infection LC treatments produced mass mortality at 30°C (median 90% mortality). Median values of the raw data (Figure S7) are given by crosses. Points are connected by solid lines to show trends between constant temperatures, with a dashed connector for the heatwave treatment.

Coinfecting parasites typically produced intermediate mortality in the *srf‐2* host genotype (Figure 5), as for N2 hosts (Figure 3A). However, combining *srf‐2* genetic susceptibility to *L. celer* and extreme thermal stress (30°C) severely eroded the protective effect of coinfection. Coinfection benefited *srf‐2* hosts with 43%–57% mortality reductions at 20°C, 25°C, and heatwave (Figure 5). At 30°C, coinfection was no longer clearly protective (95% CI for coinfection‐30°C = 80.6, 86.2 and LC‐30°C = 85.3, 89.9) (Figure 5; Table S4). We considered the implications of temperature‐sensitive protection for short‐term population dynamics in Figure S9. There, we highlight the theoretical possibility for protection to increase transmission of parasites and increase net mortality if the environment later undergoes warming.

To test if mass mortality during LC infection was due to a genotype by environment interaction, we created an additional GLMM only using LC data, but both N2 and *srf‐2* genotypes (Table S5). The susceptible *srf‐2* genotype and 30°C temperature had strong positive effects on mortality, without a clear genotype by environment interaction (Table S5).

### 3.5 Temperature Modifies Single‐Parasite Virulence Across Protective Coinfection Scenarios

We used mathematical modelling to understand how contrasting disease outcomes across conditions, such as those we observed with temperature, can occur more generally. We modelled disease scenarios exemplified by three characteristics of our experimental system, (i) non‐linear, temperature‐dependent mortality, (ii) environmental transmission of parasites and (iii) coinfection producing intermediate mortality. As the exact mechanism of coinfection protection is unclear, we considered two possible routes to lower mortality, either reduced transmission (exclusion) or reduced virulence (attenuation). We specified temperature‐dependent levels of single‐infection mortality based on our experiments (mean mortality per temperature, per *Leucobacter* species) and made a simplifying assumption that protection strength was independent of temperature.

Coinfection highlighted a consistently important role for virulence in moving between temperature‐dependent mortality levels (Figure 6; Appendix S2). When parasites excluded the competitor species, virulence was necessary and sufficient to traverse the theoretical mortality landscape for both parasites (Figure 6A,B). When coinfection attenuated disease, mortality due to ‘Parasite 1’ (exemplified by LC treatment) was also explained best by virulence (Figure 6C). Transmission only plausibly drove all observed mortality changes for ‘Parasite 2’ (exemplified by LM) in the attenuation case (Figure 6D).

**FIGURE 6:**
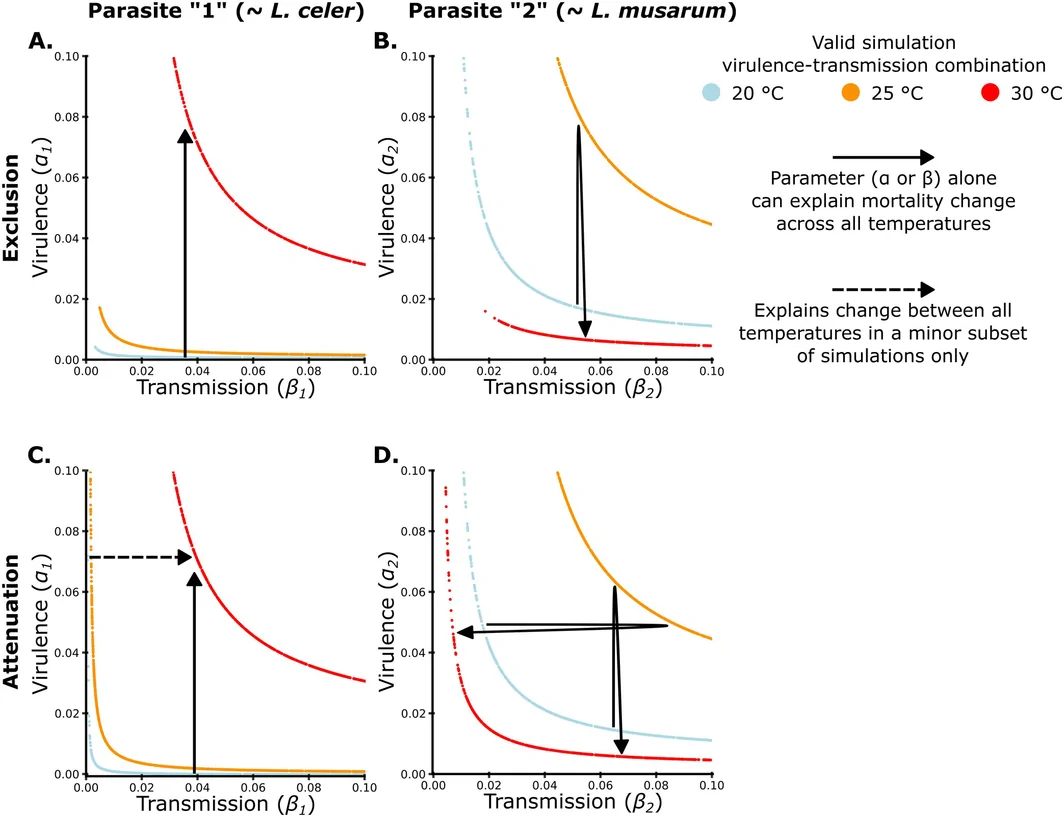
Virulence was always sufficient to shift single‐infection mortality across temperatures when parasites competed. Protective parasite competition, which limited population‐level host mortality to intermediate levels, constrained the ways by which changes in transmission or virulence could traverse the theoretical mortality landscape (Figure 1). Virulence was always sufficient to explain all shifts due to temperature, whether parasite competition excluded invasion of a second parasite (A, B) or attenuated virulence within coinfected individuals (C, D) The contours (each point is a simulation) for three levels of single infection mortality were constructed using mean mortality values taken from our experimental data, as a representative case. We applied our general model to these experimental mortality values found with *L. celer* (as ‘Parasite 1’) and *L. musarum* (as ‘Parasite 2’). Simulations were filtered such that cases in which parasite competition produced intermediate mortality were plotted, producing the truncated single‐infection mortality curves displayed. The black arrows denote how virulence or transmission changes can most simply traverse the mortality landscape from 20°C to 30°C. Possibilities exist for combined transmission and virulence changes, but we show the simplest cases, requiring only one to change. The dashed arrow in (C.) indicates transmission could explain all mortality changes, but only for very limited parameter values (4/1000 simulations at 20°C).

## 4 Discussion

Coinfection and temperatures can independently contribute to variation in disease outcomes in the wild (Hoarau et al. 2020; Paull and Johnson 2014; Pedersen and Fenton 2007). We found that temperature had disparate impacts on host mortality and parasite loads during single‐infections. When parasites competed in coinfection, however, host mortality was buffered from otherwise dramatic changes driven by the combination of heating and disease.

In ‘thermal mismatches’ when the host experiences more thermal stress than its parasites, disease can become exacerbated (Cohen et al. 2017, 2019, 2020; Sauer et al. 2020). However, we observed distinct virulence outcomes that deviated from the simplest predictions based on thermal performance alone. Sustained high temperature (30°C) shifted a benign infection to highly virulent, and vice versa, revealing contrasting host–parasite‐environment relationships. Environmental growth of the parasites predicted a thermal advantage over hosts at 30°C, but within‐host parasite loads were either reduced or unchanged relative to the host optimum temperature (20°C). The within‐host environment, at this physiological extreme, produced deviations from expectations based on thermal mismatch or metabolic theory. Although, we found some congruence of load and virulence at intermediate temperatures (20°C and 25°C) for *L. musarum* infections (as in Hector et al. 2025). The specific ecology of parasite species (Slowik et al. 2024), variation in host immunity‐parasite interactions (Mignatti et al. 2016) and mode of transmission or biological scale (i.e., individuals or populations) (Kirk et al. 2022) have all been found to add complexity to temperature‐disease interactions.

We found thermal stress introduced such complexity when crossing from physiological to extreme temperatures for the host. Thermal stress and immunity are linked at the molecular level in *C. elegans* (Feder and Hofmann 1999; Hsu et al. 2003; Singh and Aballay 2006) and, at 30°C, *C. elegans* experiences reproduction‐halting levels of stress (Begasse et al. 2015; Gouvêa et al. 2015; Petrella 2014; Plagens et al. 2021). Shifts in host reproduction‐defence trade‐offs (i.e., dynamic energy budgets) possibly explained the divergent disease outcomes we observed. For example, castrating parasites can induce hypertrophy (somatic investment) in their hosts (Hall et al. 2007) and germline‐free *C. elegans* better survive bacterial infections (Alper et al. 2009; Miyata et al. 2008; Tekippe and Aballay 2010). Host populations at reproduction‐limiting temperatures may also be poised to collapse below the required ‘threshold density’ to maintain parasite populations long‐term, where the thermal mismatch hypothesis predictions are most robust (Lloyd‐Smith et al. 2005; Rohr and Cohen 2020). Although firm thresholds may only be rarely observed in the wild (Lloyd‐Smith et al. 2005), parasites may have limited opportunity to adapt to highly stressed hosts that are sparsely or transiently present. Variation in host and parasite responses to increasingly erratic climate conditions complicates forecasting disease outcomes in emerging disease scenarios (Bull and Ebert 2008; Ekroth et al. 2021; Vasseur et al. 2014).

Differences in virulence and transmission produce variation in disease outcomes. Although virulence and transmission can be linked by parasite fitness tradeoffs (Anderson and May 1982; De Roode et al. 2008), their relationship can also be complex and dependent on other factors (Alizon and Michalakis 2015; Handel and Rohani 2015; Silva et al. 2025). Virulence, rather than transmission, most robustly explained temperature‐dependent mortality in our mathematical models. As such, we equate changes in mortality in our experiments as changes in virulence. In contrast, other systems (e.g., tested without protective coinfections) have found temperature changes that significantly alter transmission (Ismail et al. 2024; Suh et al. 2024). Distinguishing virulence and transmission effects across temperatures is a key component of evaluating disease risks and possible interventions (Nguyen et al. 2021; Paull and Johnson 2014). Furthermore, modelling scenarios where protective coinfection is thermally sensitive (e.g., *srf‐2* mutants at 30°C) could reveal different infection dynamics, such as those that lead to mass mortality.

Coinfected hosts died less across temperatures than hosts singly‐infected with the most virulent disease. Competition reduced loads of both parasite species, effectively conferring infection resistance to hosts and likely underpinning protection. Although virulence‐load links on a per‐parasite basis had mixed results, the overall trend of coinfection reducing host mortality and loads of both parasites was clear. Transcriptomic work in this system has hypothesized that this protective coinfection may operate by exploitative competition between parasites for ‘public goods’, operating in parallel to endogenous host defences (Will et al. 2025). This parasite–parasite competition may alleviate immunity‐reproduction tradeoffs in the host resource budget (Will et al. 2025), which could offset temperature‐dependent changes in host–parasite interactions. Although temperature can alter the outcomes of microbial antagonisms (Dal Bello and Abreu 2024; Li et al. 2022), we found that these *Leucobacter* parasites were thermally matched and protection was effective across temperatures. We additionally tested mutant hosts, with inversed susceptibilities to the *Leucobacter* species (Hodgkin et al. 2013), and found coinfection remained protective. Parasite–parasite conflict can thus affect hosts across contexts—dampening the impacts of either environment (e.g., temperature) or host biology (e.g., genotype) on variation in virulence. Regardless of which parasite was the principal driver of virulence, coinfection limited host mortality and loads of both species constrained by a shared parasite carrying capacity of the host. We nevertheless demonstrated that combined environmental *and* host biology factors can nearly wipe out infected host populations, whether singly‐ or coinfected. This finding supports observations in the wild for mass mortality events being commonly driven by infectious disease, with additional stressors enhancing virulence (Carella et al. 2019; Fey et al. 2015; Sanderson and Alexander 2020). Further research could explore conditions where the parasites themselves are thermally mismatched and if different modes of coinfection interactions (e.g., exploitative competition for host nutrients, spiteful molecular warfare, cooperation, or sequential exposure) (Ramesh and Hall 2025) affect disease in novel ways. Parasite interactions across large taxonomic divides, utilizing distinct host‐resources, or interacting at a distance between host‐compartments (Erazo et al. 2025; Graham 2008; Lello et al. 2018), could capture different modes of competition than we observed between the congener species we tested.

In a warming world with parasites spreading to areas undergoing rapid heating and environmental change, temperate host species (and their local parasites) may be challenged by novel parasite threats from warmer regions (Carlson et al. 2022). Coinfecting parasites may spread in tandem (Kafle et al. 2020; Oakgrove et al. 2014). Protective coinfection could mitigate the worst possible outcomes from emerging infectious disease. As coinfections are widespread (Telfer et al. 2008), predictions based purely on single infection thermal mismatches or metabolic first principles may misestimate the impacts on animal health when not accounting for inter‐parasite interactions. Protective coinfections in particular may reduce host mortality levels in the short term but may facilitate the rapid transmission of both parasites across time. These widespread infections could be a latent source of severe disease, emerging when protection breaks down.

## Author Contributions

I.W. and K.C.K. designed the experiments. I.W. and T.E.H. collected the data. C.A.S. developed the mathematical models. I.W. conducted the statistical analyses. I.W., C.A.S., T.E.H. and K.C.K. wrote the manuscript.

## Funding

This work was supported by the European Research Council (802242), the Natural Environment Research Council (NE/X000540/1), and an NSERC Canada Excellence Research Chair.

## Conflicts of Interest

The authors declare no conflicts of interest.

## Supporting information


**Data S1:** Excel file with posterior estimates for the N2 host mortality model across reference levels.

**Table S1:** A model of *L. celer* loads indicated that bacterial abundance was reduced both by coinfection and constant high temperature (30°C).
**Table S2:** A model of *L. musarum* loads indicated that bacterial abundance was maximal at 25°C and suppressed by coinfection except during heatwave.
**Table S3:** A model of host mortality indicated clear temperature, infection and interactive effects.
**Table S4:** A model of *srf‐2* host mortality found that coinfection interactions continued to protect hosts, even has temperature drove large changes in mortality.
**Table S5:** A model of LC host mortality indicated clear genotype and temperature effects, but their interaction did not drive mass mortality.
**Figure S1:** Using a fully factorial design, we analysed up to seven batches of N2 genotype infection assays to produce the primary dataset.
**Figure S2:** Nematode washing removed the vast majority of bacteria not directly associated with the host.
**Figure S3:** Parasite coinfection competition mediated by the host appears to be stronger than environmental competition alone.
**Figure S4:** Maximum growth rate observed in single cultures of *Leucobacter* grown in vitro at different temperatures.
**Figure S5:** Parasite fitness (CFU load) was generally reduced by coinfection, with secondary effects of temperature.
**Figure S6:** Coinfecting parasite interactions modified temperature‐dependent host mortality.
**Figure S7:** Parasite interactions persisted to give intermediate mortality, despite major changes in host condition due to genetic susceptibility.
**Figure S8:** Bacterial competition is typically mutually harmful.
**Figure S9:** Increased survivorship due to protection could lead to greater parasite transmission and downstream increases in deaths.
**Appendix S1:** Host fecundity is primarily shaped by temperature and secondarily by infection.
**Appendix S2:** Mathematical models.
**Appendix S3:** Severe warming may link coinfecting parasite loads with virulence and protection.

## Data Availability

The accompanying code for the mathematical mode can be found at: https://doi.org/10.5281/zenodo.17897997.
